# Age- and gender-related prevalence of multimorbidity in primary care: the swiss fire project

**DOI:** 10.1186/1471-2296-13-113

**Published:** 2012-11-24

**Authors:** Alessandro Rizza, Vladimir Kaplan, Oliver Senn, Thomas Rosemann, Heinz Bhend, Ryan Tandjung

**Affiliations:** 1 Division of Internal Medicine, University Hospital Raemistrasse 100, Zurich, CH-8091, Switzerland; 2 Institute of General Practice and Health Services Research, University of Zurich, Zurich, Switzerland

**Keywords:** Multimorbidity, Chronic medical conditions, Prevalence, Primary care, Age, Gender, Swiss, FIRE

## Abstract

**Background:**

General practitioners often care for patients with several concurrent chronic medical conditions (multimorbidity). Recent data suggest that multimorbidity might be observed more often than isolated diseases in primary care. We explored the age- and gender-related prevalence of multimorbidity and compared these estimates to the prevalence estimates of other common specific diseases found in Swiss primary care.

**Methods:**

We analyzed data from the Swiss FIRE (Family Medicine ICPC Research using Electronic Medical Record) project database, representing a total of 509,656 primary care encounters in 98,152 adult patients between January 1, 2009 and July 31, 2011. For each encounter, medical problems were encoded using the second version of the International Classification of primary Care (ICPC-2). We defined chronic health conditions using 147 pre-specified ICPC-2 codes and defined multimorbidity as 1) two or more chronic health conditions from different ICPC-2 rubrics, 2) two or more chronic health conditions from different ICPC-2 chapters, and 3) two or more medical specialties involved in patient care. We compared the prevalence estimates of multimorbidity defined by the three methodologies with the prevalence estimates of common diseases encountered in primary care.

**Results:**

Overall, the prevalence estimates of multimorbidity were similar for the three different definitions (15% [95%CI 11-18%], 13% [95%CI 10-16%], and 14% [95%CI 11-17%], respectively), and were higher than the prevalence estimates of any specific chronic health condition (hypertension, uncomplicated 9% [95%CI 7-11%], back syndrome with and without radiating pain 6% [95%CI 5-7%], non-insulin dependent diabetes mellitus 3% [95%CI 3-4%]), and degenerative joint disease 3% [95%CI 2%-4%]). The prevalence estimates of multimorbidity rose more than 20-fold with age, from 2% (95%CI 1-2%) in those aged 20–29 years, to 38% (95%CI 31-44%) in those aged 80 or more years. The prevalence estimates of multimorbidity were similar for men and women (15% vs. 14%, p=0.288).

**Conclusions:**

In primary care, prevalence estimates of multimorbidity are higher than those of isolated diseases. Among the elderly, more than one out of three patients suffer from multimorbidity. Management of multimorbidity is a principal concern in this vulnerable patient population.

## Background

Advances in medical science and technology allow turning formerly fatal episodes of acute diseases into survivable events, often resulting in chronic health conditions [1]. A chronic health condition is a term that includes both, chronic diseases that require ongoing medical care, and persisting impairments (deficit in vision or hearing, orthopedic impairment) that limit the person’s functionality [2,3]. Estimates from the US suggest that by 2020 nearly 50% of the population will have at least one chronic health condition [4], with most of these patients suffering from other concurrent chronic health conditions.

Even though primary care physicians treat multimorbid patients on a daily basis, valid figures about prevalence of multimorbidity are scarce [5]. Studies from the United States and Canada [6-8], Australia [9], and several European countries [10,11] report an increasing prevalence of multimorbidity with age, but the figures vary widely due to different patient populations, study settings, and definitions of multimorbidity (i.e., the medical conditions taken into account and the number of medical condition required to define multimorbidity) [6,9-11]. In Switzerland, figures regarding prevalence of multimorbidity in primary care settings are entirely lacking.

In view of the growing importance of multimorbidity, we conducted a retrospective cohort analysis in primary care practices in the German speaking part of Switzerland with the aim to explore the age- and gender-related prevalence of multimorbidity and to compare these estimates with the prevalence estimates of other common specific diseases found in Swiss primary care. We hope that our results will initiate a discussion, about how future primary care should be provided, particularly among the elderly, in the most effective way.

## Methods

### Data source and data extraction

We obtained primary care based data from the Swiss FIRE (Family Medicine ICPC Research using Electronic Medical Record) project, which was initiated in the year 2009 by the SGAM (Association of Swiss General Practitioners) in collaboration with the Institute of General Practice at the University of Zurich [12]. Primary care physicians practicing in the German speaking part of Switzerland provided voluntarily standardized data on all patient-physician encounters from the beginning of January 2009 to the end of July 2011. Data exporter software extracted administrative data, patient’s demographics, vital signs, diagnostic codes, laboratory values, and medication from the electronic patient records, de-identified the data sets, and uploaded the anonymized information on a central server. Only those medical conditions addressed during any encounter were coded. Further analysis of the anonymized data was allowed according to the Swiss data protection regulation without explicit consents of patients.

### Cohort construction

First, we excluded all patients with missing information on age or gender. Second, we excluded all patients younger than 20 years. Third, we excluded all those encounters, for which no diagnostic codes were provided (administrative visits like phone calls and provision of a prescription, where the patient was not seen by a physician and codes for the encounter were not available).

### Disease classification

We classified morbidity according to the International Classification in Primary Care - 2nd version (ICPC-2) [13]. ICPC-2 is a morbidity classification system designed for primary care, developed by the World Organization of Family Doctors (Wonca). The classification system is structured in 17 chapters and seven components, which remain the same for each chapter. ICPC-2 encompasses 686 symptoms and diagnostic rubrics.

### Definition of chronic health conditions

We defined chronic health conditions based on the ICPC-2 classification system using the concept of O`Halloran et al., which considers both, specific chronic diagnoses or chronic symptoms and health complains [3]. A total of 147 (21.4%) codes from the 686 ICPC-2 codes were identified as chronic conditions. The full list of the ICPC-2 rubrics classified as chronic is provided as supplementary data of the original paper at http://www.fmrc.org.au/classifi.htm.

### Definition of multimorbidity

We used three different methods to define multimorbidity as present: 1) two or more chronic health conditions referring to different rubrics of the ICPC-2 classification system; 2) two or more chronic health conditions referring to different chapters of the ICPC-2 classification system; 3) and two or more medical specialties involved in the care of chronic health conditions in one patient (i.e., stroke refers to the nervous system and not the cardiovascular system, and is managed by the neurologist and not the cardiologist). A complete classification of chronic health conditions by medical specialties is provided in Additional file 1.

### Statistical analysis

The study had a cluster sample design, and we adjusted for the clusters in the analyses. We explored the prevalence estimates of common chronic health conditions and compared these estimates to those of multimorbidity as defined by the three different methods. We provide the prevalence estimates of chronic health conditions and multimorbidity stratified by age and gender. We report counts and proportions and the 95% confidence intervals and use a multilevel mixed-effect logistic regression for comparison. We assumed a significance level at a p-value <0.001 because of large sample size and multiple comparisons. We managed data and conducted analyses using Stata® Version 11.2 (Stata Corporation, College Station, TX, USA; http://www.stata.com).

## Results

### Cohort selection

**Figure 1:**
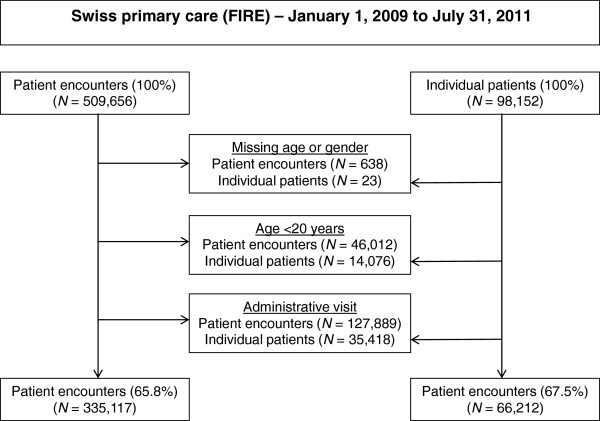
**Selection of the study cohorts.** All patient-physician encounters extracted from the Swiss primary care database FIRE (Family Medicine ICPC Research using Electronic Medical Records) between January 1, 2009 and July 31, 2011 were eligible.

The detailed study cohort selection is depicted in Figure 1. From the central server 509,656 patient-physician encounters in 98,152 individual patients and 64 individual physicians were extracted between January 1, 2009 and July 31, 2011. We dropped 635 encounters in 20 individual patients because of missing gender and 3 encounters in 3 individual patients because of missing age. Further, we dropped 46,012 encounters in 14,076 individual patients because the age at the time of last encounter was less than 20 years. Further, we excluded all encounters taking place only because of administrative reasons and therefore lacking any ICPC-2-codes. Finally, 335,117 encounters in 66,212 individual patients and 63 individual physicians remained for analysis.

### Baseline characteristics

The number of patients and the number of consultations per physician varied from 3 to 2,669 and from 3 to 20,841, respectively. The median number of physician encounters per patient was 11 (range 1–242). The mean (+SD) age at last physician encounter was 49.6 (+19.2) years (range 20 to 111 years). Slightly more than half of the study cohort were females (52.8% vs. 47.2%, p<0.001). Women were slightly older than men (50.2 vs. 48.9 years, p<0.001).

### Chronic health conditions

We identified 44,619 chronic health conditions among 66,212 adult patients (>20 years). The prevalence of common (prevalence >1%) chronic health conditions is provided in Table 1. The most common chronic health conditions found were “hypertension, uncomplicated (K86)” (9.37% [95%CI 7.40-11.35%]), “diabetes, non-insulin dependent (T90)” (3.29% [95%CI 2.75-3.82%]), “back syndrome without radiating pain (L84)” (3.20% [95%CI 2.17-4.23%]), “back syndrome with radiating pain (L86)” (2.75% [95%CI 1.85-3.64%]), “lipid disorders (T93)” (2.75% [95%CI 1.49-4.00%]), “depressive disorder (P76)” (2.40% [95%CI 1.96-2.84%) and “obesity (T82)” (2.38% [95%CI 1.42-3.34%]). All circulatory chronic conditions except “hypertension, uncomplicated” had prevalence estimates below 2%.

**Table 1:** Prevalence estimates of common chronic health conditions* **among 66**,**212 adult patients** (**age** ≥**20 years**) **in Swiss primary care**

**Chronic health condition(ICPC**-2)*	**All(%)**	**Men(%)**	**Women(%)**	**p**-**value**
Circulatory				
Ischemic heart disease without angina (K76)	1.09	1.73	0.52	<.001
Atrial fibrillation/flutter (K78)	1.45	1.63	1.30	0.001
Hypertension, uncomplicated (K86)	9.37	9.78	9.01	0.037
Varicose veins of leg (K95)	1.16	0.78	1.50	<.001
Musculoskeletal (Locomotion)				
Neck syndrome (L83)	1.35	1.23	1.46	0.050
Back syndrome without radiating pain (L84)	3.20	3.32	3.09	0.142
Back syndrome with radiating pain (L86)	2.75	2.67	2.84	0.249
Osteoarthritis of knee (L90)	1.49	1.27	1.69	0.001
Shoulder syndrome (L92)	1.58	1.52	1.64	0.346
Osteoporosis (L95)	1.01	0.27	1.68	<.001
Psychological				
Depressive disorder (P76)	2.40	1.75	3.00	<.001
Respiratory				
Asthma (R96)	1.54	1.39	1.67	0.005
Skin				
Dermatitis/atopic eczema (S87)	1.26	1.27	1.25	0.918
Endocrine, metabolic and nutritional				
Obesity (T82)	2.37	2.42	2.36	0.663
Diabetes, non-insulin dependent (T90)	3.29	4.04	2.63	<.001
Lipid disorder (T93)	2.75	3.25	2.3	<.001

ICPC-2=International Classification in Primary Care-2nd version.* Chronic health conditions were defined based on the ICPC-2 classification system using the concept of O`Halloran et al. [3]. Chronic health conditions with a prevalence >1% are listed.

More than one third of the patients had one or more chronic health conditions (38.0% [95%CI 33.6-42.4%]) (Table 2). A single chronic health condition was found in 15,526 patients (23.5% [95%CI 21.6-25.3%]), two chronic health conditions were found in 4,790 patients (7.23% [95%CI 6.06-8.41%]), three chronic health conditions were found in 2,319 (3.50% [95%CI 2.73-4.28%]), and more than three chronic health conditions were found in 2,513 patients (3.80% [95%CI 2.31-5.28%]). Two patients presented with 14 chronic health conditions. The proportion of patients with one or more chronic health condition increased with age from 15.6% (95%CI 13.5-17.7%) for those in the youngest age group (20–29 years) to 68.4% (95%CI 63.9-72.9%) for those in the oldest age group (>80 years) (Table 2). Overall, the proportion of patients with one or more chronic health condition was similar in men and women (38.3% vs. 37.7%, p=0.585). However, stratified on age groups, the proportion of elderly with one or more chronic health condition was higher for men than women (58.3% vs. 53.0% for those aged 60–69 years, p<0.001; and 68.3% vs. 62.3% for those aged 70–79 years, p<0.001) (Table 2).

**Table 2:** **Prevalence estimates of one or more**, **two or more**, **and three or more chronic health conditions** (**ICPC**-**2**)* **among 66**,**212 adult patients** (**age** ≥**20 years**) **in swiss primary care stratified by age**- **and gender**

	**All**		**Men**		**Women**		**p**-**value**
	**%**	**(95%CI)**	**%**	**(95%CI)**	**%**	**(95%CI)**	
**One or more chronic health conditions**							
All	38.0	(33.6-42.4)	38.3	(34.1-42.4)	37.7	(32.9-42.5)	0.585
Age group (years)							
20–29	15.6	(13.5-17.7)	15.0	(12.8-17.2)	16.1	(13.7-18.5)	0.244
30–39	19.8	(16.0-23.5)	19.5	(15.9-23.2)	20.0	(15.9-24.1)	0.655
40–49	32.2	(28.0-36.3)	31.7	(27.4-35.9)	32.6	(28.1-37.2)	0.487
50–59	43.9	(39.2-48.5)	45.4	(40.7-50.0)	42.4	(37.4-47.3)	0.102
60–69	55.5	(50.8-60.3)	58.3	(53.8-62.6)	53.0	(47.6-58.2)	<.001
70–79	65.0	(60.2-69.8)	68.3	(63.3-73.2)	62.3	(57.4-67.2)	<.001
>80	68.4	(63.9-72.9)	70.3	(66.0-74.6)	67.3	(62.5-72.0)	0.096
**Two or more chronic health conditions**							
All	14.5	(11.4-17.7)	14.8	(11.6-18.0)	14.3	(11.1-17.5)	0.288
Age group (years)							
20–29	1.70	(1.11-2.29)	1.70	(1.11-2.29)	2.12	(1.28-2.96)	0.182
30–39	3.28	(2.12-4.44)	3.31	(2.08-4.53)	3.26	(2.07-4.44)	0.560
40–49	8.16	(6.21-10.1)	8.18	(6.15-10.2)	8.15	(6.08-19.2)	0.650
50–59	15.7	(12.3-19.1)	16.5	(12.8-20-2)	14.8	(11.6-18.1)	0.207
60–69	25.8	(20.9-30.8)	28.8	(23.6-33.9)	22.9	(18.1-27.8)	<.001
70–79	33.6	(27.2-40.0)	35.6	(28.5-42.6)	31.9	(25.9-37.9)	0.030
>80	37.7	(31.2-44.2)	38.9	(32.0-45.7)	37.0	30.6-43.5)	0.643
**Three or more chronic health conditions**							
All	7.30	(5.12-9.48)	7.55	(5.23-9-87)	7.07	(4.97-9.17)	0.952
Age group (years)							
20–29	0.42	(0.22-0.62)	0.40	(0.13-0.67)	0.44	(0.21-0.66)	0.515
30–39	0.95	(0.54-1.35)	1.01	(0.53-1.48)	0.90	(0.49-1.31)	0.860
40–49	2.69	(1.79-3.59)	2.67	(1.71-3.61)	2.72	(1.76-3.68)	0.720
50–59	6.82	(4.71-8.92)	7.71	(5.18-10.2)	5.91	(4.10-7.71)	0.055
60–69	13.0	(9.35-16.7)	14.4	(10.4-18.4)	11.7	(8.25-15.1)	0.002
70–79	20.0	(14.3-25.6)	21.9	(15.3-28.5)	18.4	(13.3-23.4)	0.007
>80	22.7	(16.8-28.5)	24.0	(17.3-30.7)	21.9	(16.4-27.4)	0.283

ICPC-2=International Classification in Primary Care-2nd version.* Chronic health conditions were defined based on the ICPC-2 classification system using the concept of O`Halloran et al. [3].

### Multimorbidity

For the entire study cohort, the prevalence estimate of multimorbidity defined as two or more chronic health conditions from different ICPC-2 classification rubrics was 14.5% (95%CI 11.4-17.7%) (Table 2) and was higher than the prevalence estimate of the most common specific chronic health condition found (hypertension, uncomplicated: 9.37% [95%CI 7.40-11.35%]; p<0.001). The prevalence of multimorbidity rose more than 20-fold with age, and was 1.70% (95%CI 1.11-2.29%) for those in the youngest age group (20–29 years), and 37.7% (95%CI 31.3-44.2%) for those in the oldest age group (>80 years) (Table 2). Overall, the prevalence of multimorbidity was similar for men and women (14.8 vs. 14.3%, p=0.288). However, stratified on age groups, the proportion of elderly with multimorbidity was higher for men than women (28.8% vs. 22.9% for those aged 60–69 years, p<0.001; and 35.6% vs. 31.9% for those aged 70–79 years, p=0.030) (Table 2).

### Comparison of different definitions of multimorbidity

**Figure 2:**
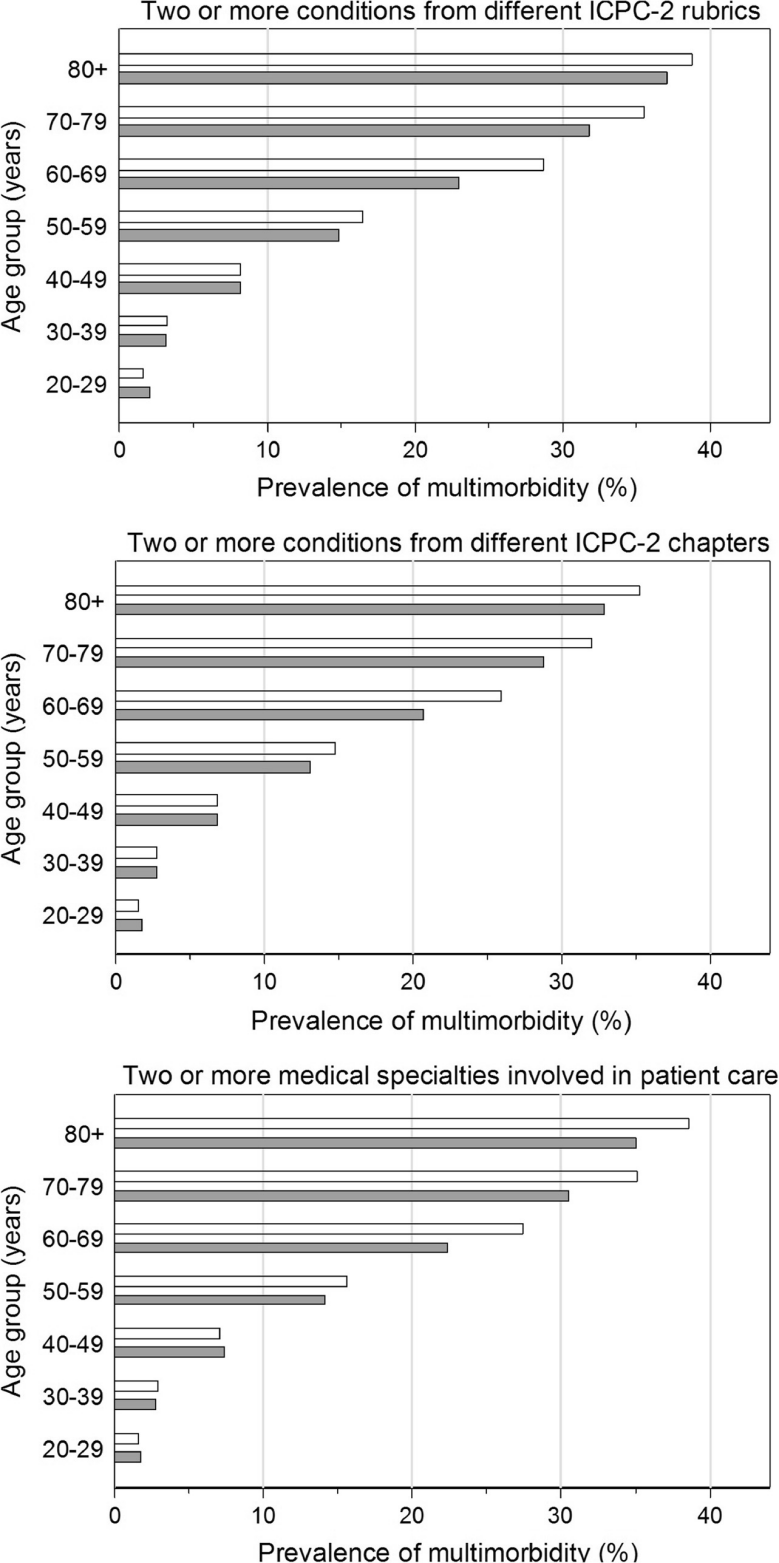
**Age- and gender-related prevalence estimates of multimorbidity based on three different definitions.** The upper panel presents estimates of multimorbidity defined as two or more chronic health conditions from different ICPC-2 rubrics, the middle panel those defined as two or more chronic health conditions from different ICPC-2 chapters, and the lower panel those defined as involvement of two or more medical specialties. On the x-axis, prevalence of multimorbidity is presented. On the y-axis, age is stratified in seven age groups. grey bars represent the estimates for women and white bars those for men.

Overall, the prevalence estimates of multimorbidity for the three different definitions of multimorbidity, i.e., two or more chronic health conditions referring to different rubrics of the ICPC-2 classification system, two or more chronic health conditions referring to different chapters of the ICPC-2 classification system, and two or more specialties involved in the medical care of chronic health conditions in one patient, were similar with 14.5% (95%CI 11.4-17.7%), 13.0% (95%CI 10.1-15.8%), and 13.8% (95%CI 10.9-16.8%), respectively. The increase in prevalence of multimorbidity with age was similar for the three different definitions of multimorbidity (Figure 2). The prevalence estimates of multimorbidity were similar for men and women independent of the definitions of multimorbidity used (14.8% vs. 14.3%, p=0.185; 13.3% vs. 12.7%, p=0.350; 14.2% vs. 13.5%, p=0.540).

## Discussion

Our study revealed that multimorbidity (defined as two or more concurrent chronic health condition in one patient) is more often observed than isolated diseases in Swiss primary care. Multimorbidity was 1.5 times as prevalent (14.5% [95%CI 11.4-17.7%]) as the most common single specific chronic medical condition (hypertension: 9.37% [95%CI 7.40-11.35%]). Without restricting our analysis to chronic medical conditions only, the prevalence estimates of multimorbidity would have been even higher. As expected, prevalence estimates of multimorbidity increased with age, rising more than 20-fold from the youngest (1.70% [1.11-2.29]) to the oldest age group (37.7 [31.2-44.2]) and affecting more than one out of three elderly patients. Although we found similar gender-specific prevalence of multimorbidity for the entire study population, stratified on age groups, elderly women had a prevalence estimates of multimorbidity close to that of men ten years younger (Table 2).

Estimates of prevalence of multimorbidity vary widely in the literature, related to different definitions of multimorbidity (type and number of diseases included in the definition), diverse data sources (questionnaires, medical records, patient charts, and administrative data), and dissimilar patient populations studied [2,14], making comparisons between studies difficult. However, most estimates from primary care, which relied on practitioners records as data source, reported prevalence rates between 20-30% for the entire population, and 50-90% for the elderly [6,10,11,15,16], which is roughly twice the prevalence found in our data. The most obvious reason for this low prevalence of multimorbidity in our study is under-coding of chronic health conditions. The participating general practitioners in the FIRE project were initially instructed to code symptoms or diagnoses, which were addressed during consultations or needed special attention, such as a drug prescription. Therefore, chronic conditions such as obesity, hearing loss, or visual impairment, which were not addressed during a patient-physician encounter, were not coded. By accumulating all chronic health conditions coded in an individual patient across the entire observation period, we were able to raise the prevalence rates for specific chronic conditions. However, under-coding remained a problem. For example, based on the ICPC-2 codes T89 (insulin-dependent diabetes mellitus) and T 90 (non-insulin dependent diabetes mellitus), we found an overall prevalence estimate of diabetes mellitus of 3.96% (95%CI 3.22%-4.39%), yet including those patients with a blood glucose >11.1 mmol/L or glycosylated hemoglobin >6.5% (diabetes by definition) and those prescribed anti-diabetic medication, the prevalence rose to 5.02% (95%CI 4.26%-5.88%). Nonetheless, such an internal coding validation was not feasible for all 147 chronic health conditions included in our definition of multimorbidity, therefore, we report our estimates based on ICPC-2 codes alone, acknowledging significant under-coding resulting in an underestimation of the burden of disease.

Not surprisingly, our results confirm the striking age-dependent increase in prevalence of multimorbidity observed in many European [10,11,17] and North American [6,8] studies, a pattern that results due to accumulation of chronic health conditions during the ageing process. The presence of this pattern independent of the definition of multimorbidity used (Figure 2), is reassuring regarding the consistency of our data considering the significant under-coding discussed above.

It is proven that men in industrialized countries die earlier than women, but that women have poorer health than men [18]. This might be possibly due to more illnesses and disabilities in women, which are not life-threatening, and more serious and often deadly conditions in men [18]. As expected, our data show a higher prevalence estimates of potentially deadly chronic health conditions in men (cardiovascular disease, diabetes, and lipid disorders), whereas women suffer more often from less “deadly” chronic conditions (varicose veins, osteoarthritis, osteoporosis, and depression) (Table 1). Conversely, after adjusting for age, we found higher prevalence estimates of multimorbidity in elderly men, which is opposite to common belief and not supported by some recent publications [11,17,19]. However, this gender effect was not consistent across all studies [19,20] and conclusions should be inferred with caution. A specific concern regarding our data that might have contributed to the observed gender effect is under-coding of gynecological problems (many women in Switzerland see their gynecologists independent of consultations with their general practitioners), and under-coding of non-life-threatening chronic health conditions.

Multimorbidity was observed in all age groups, and not only in the elderly, a fact that has been shown in previous studies [11]. This observation can be explained, because young patients are both, very healthy and not seen by primary care physicians at all, or they have serious diseases with co-existing related medical conditions and present with multimorbidity.

Our study has several strengths and limitations. The main strength of our study is a large, robust data set, generated by practitioners trained in coding using the ICPC-2 coding system, which is a validated ambulatory coding system for primary care that includes impairments (e.g., hearing impairment). Limitations are: First, the relative small number of participating practices (63) may reduce the generalizability of our results to the Swiss primary care. Second, our data capture the actual prevalence estimates of multimorbidity in the population that consults general practitioners, and not the prevalence estimates in the general population. Third, our definition of multimorbidity might seem quite arbitrary, because a simple count of chronic condition lacks the information of a specific impact on the health system, e.g., a diabetic patient with coronary heart disease has not the same impact on resources use as an obese patient with hearing impairment; however, because we lacked information about the severity of the conditions, we could not use more precise instruments. Forth, the proportion of elderly with one or more chronic health condition was higher for men than women (Table 2) using the chi square statistic, however, no significant differences between sexes were found using 95% confidence intervals. This was due to wide degree of uncertainty in each estimate because of the cluster sample design. Fifth, the potentially severe underestimation because of under-coding was already discussed above.

## Conclusion

We conclude that multimorbidity is observed more often than isolated diseases in Swiss primary care. It is more common than any single specific chronic health condition. Prevalence of multimorbidity markedly increases with age. More than one out of three elderly patients suffer from multimorbidity. However, prevalence estimates, based on a disease-count methods during physician-patient encounters, as done in this study, warrant further confirmation using more accurate data collection methods.

Guidelines focusing on multimorbidity are badly needed to help general practitioners coping with the rising complexity of care in the vulnerable patient population with multimorbidity. Primary care research networks are excellent resources for better understanding the type of relationships existing between co-occurring diseases. The FIRE project is a novel approach in Switzerland that tries to quantify the burden of chronic diseases and multimorbidity in Swiss primary care, in order to help detecting and understanding specific targets for effective future intervention in patients with multimorbidity.

## Abbreviations

ICPC-2: International Classification of primary Care second edition; FIRE: Family Medicine ICPC Research using Electronic Medical Record.

## Competing interests

The authors declare that they have no competing interests.

## Authors' contributions

AR made the first draft of the paper. VK extracted the figures from the database. OS help to elaborate on the draft of the manuscript. TR initiated the project and developed the ideas. HB coordinated the data collection in a central data base. RT revised the manuscript and was responsible for the final version. All authors gave their final approval of the version to be published.

## Authors’ information

Members of the FIRE study group, Aargau, Switzerland.

## Support

Unconditional funding from the Swiss Association of General Practitioners (SGAM).

## Pre-publication history

The pre-publication history for this paper can be accessed here:

http://www.biomedcentral.com/1471-2296/13/113/prepub

## Supplementary Material

Additional file 1 **Appendix.** Chronic health conditions* grouped by medical specialties^†^. Click here for file

## References

[B1] Clarke A What is a chronic disease? the effects of a re-definition in HIV and AIDS Soc Sci Med 1994 39 4 591 597 10.1016/0277-9536(94)90102-3 7973859

[B2] Perrin EC Newacheck P Pless IB Drotar D Gortmaker SL Leventhal J Perrin JM Stein RE Walker DK Weitzman M Issues involved in the definition and classification of chronic health conditions Pediatrics 1993 91 4 787 793 8464668

[B3] O'Halloran J Miller GC Britt H Defining chronic conditions for primary care with ICPC-2 Fam Pract 2004 21 4 381 386 10.1093/fampra/cmh407 15249526

[B4] Wu SY Green A: Projection of chronic illness prevalence and costs inflation 2000 Santa Monica: RAND Health

[B5] Fortin M Lapointe L Hudon C Vanasse A Multimorbidity is common to family practice: is it commonly researched? Can Fam Physician 2005 51 244 245 16926936 PMC1472978

[B6] Fortin M Bravo G Hudon C Vanasse A Lapointe L Prevalence of multimorbidity among adults seen in family practice Ann Fam Med 2005 3 3 223 228 10.1370/afm.272 15928225 PMC1466875

[B7] Fortin M Hudon C Haggerty J Akker M Almirall J Prevalence estimates of multimorbidity: a comparative study of two sources BMC Health Serv Res 2010 10 111 10.1186/1472-6963-10-111 20459621 PMC2907759

[B8] Wolff JL Starfield B Anderson G Prevalence, expenditures, and complications of multiple chronic conditions in the elderly Arch Intern Med 2002 162 20 2269 2276 10.1001/archinte.162.20.2269 12418941

[B9] Britt HC Harrison CM Miller GC Knox SA Prevalence and patterns of multimorbidity in Australia Med J Aust 2008 189 2 72 77 18637770 10.5694/j.1326-5377.2008.tb01919.x

[B10] van den Akker M Buntinx F Metsemakers JF Roos S Knottnerus JA Multimorbidity in general practice: prevalence, incidence, and determinants of co-occurring chronic and recurrent diseases J Clin Epidemiol 1998 51 5 367 375 10.1016/S0895-4356(97)00306-5 9619963

[B11] Uijen AA van de Lisdonk EH Multimorbidity in primary care: prevalence and trend over the last 20 years Eur J Gen Pract 2008 14 Suppl 1 28 32 18949641 10.1080/13814780802436093

[B12] Chmiel Moshinsky C Bhend H Senn O Zoller M Rosemann T The FIRE project: a milestone for research in primary care in Switzerland Swiss Med Wkly 2011 140 w13142 21279858 10.4414/smw.2011.13142

[B13] ICPC-2-R International Classification of Primary Care. Revised Second Edition 2005 Oxford: Oxford University Press

[B14] De Groot V Beckerman H Lankhorst GJ Bouter LM How to measure comorbidity: a critical review of available methods J Clin Epidemiol 2003 56 3 221 229 10.1016/S0895-4356(02)00585-1 12725876

[B15] Schneider KM O'Donnell BE Dean D Prevalence of multiple chronic conditions in the United States' Medicare population Health Qual Life Outcomes 2009 7 82 10.1186/1477-7525-7-82 19737412 PMC2748070

[B16] Marengoni A Angleman S Melis R Mangialasche F Karp A Garmen A Meinow B Fratiglioni L Ageing with multimorbidity: a systematic review of the literature Ageing Res Rev 2011 10 4 430 439 10.1016/j.arr.2011.03.003 21402176

[B17] Laux G Kuehlein T Rosemann T Szecsenyi J Co- and multimorbidity patterns in primary care based on episodes of care: results from the German CONTENT project BMC Health Serv Res 2008 8 14 10.1186/1472-6963-8-14 18205916 PMC2244601

[B18] Macintyre S Hunt K Sweeting H Gender differences in health: are things really as simple as they seem? Soc Sci Med 1996 42 4 617 624 10.1016/0277-9536(95)00335-5 8643986

[B19] Marengoni A Winblad B Karp A Fratiglioni L Prevalence of chronic diseases and multimorbidity among the elderly population in Sweden Am J Public Health 2008 98 7 1198 1200 10.2105/AJPH.2007.121137 18511722 PMC2424077

[B20] Glynn LG Valderas JM Healy P Burke E Newell J Gillespie P Murphy AW The prevalence of multimorbidity in primary care and its effect on health care utilization and cost Fam Pract 2011 28 5 516 523 10.1093/fampra/cmr013 21436204

