# Supplementary material for: Age- and gender-related prevalence of multimorbidity in primary care: the swiss fire project
Source: BMC Fam Pract. 2012 Nov 24;13:113. doi: 10.1186/1471-2296-13-113 (PMC3557138; doi:10.1186/1471-2296-13-113)
Supplement: Additional file 1 — Appendix. Chronic health conditions* grouped by medical specialties†. [file 1471-2296-13-113-S1.doc]

Appendix

Chronic health conditions* grouped by medical specialties^†^.

| Infectious diseases |
| --- |
| Tuberculosis (A70) |
| HIV-infection/AIDS (B90) |
| Neurological infection other (N73) |
| Oncology |
| Malignancy NOS (A79) |
| Hodgkin’s disease/lymphoma (B72) |
| Leukemia (B73) |
| Malignant neoplasm blood other (B74) |
| Malignant neoplasm stomach (D74) |
| Malignant neoplasm colon/rectums (D75) |
| Malignant neoplasm pancreas (D76) |
| Malignant digestive neoplasm other(D77) |
| Malignant neoplasm nervous system (N74) |
| Malignant neoplasm bronchus/lung (R84) |
| Malignant neoplasm respiratory other (R85) |
| Malignant neoplasm thyroid (T71) |
| Malignant neoplasm of kidney (U75) |
| Malignant neoplasm of bladder (U76) |
| Malignant neoplasm urinary other (U77) |
| Malignant neoplasm breast female (X76) |
| Malignant neoplasm genital female other (X77) |
| Malignant neoplasm prostate (Y77) |
| Malignant neoplasm male genital other (Y78) |
| Hematology |
| Hereditary hemolytic anemia (B78) |
| Anemia vit B12/folate deficiency (B81) |
| Purpura/coagulation defect (B83) |
| Gastroenterology |
| Malignant neoplasm stomach (D74) |
| Malignant neoplasm colon/rectum (D75) |
| Malignant neoplasm pancreas (D76) |
| Malignant digestive neoplasm other/nos (D77) |
| Esophagus disease (D84) |
| Duodenal ulcer (D85) |
| Peptic ulcer other (D86) |
| Diverticular disease (D92) |
| Irritable bowel syndrome (D93) |
| Chronic enteritis/ulcerative colitis (D94) |
| Liver disease nos (D97) |
| Cholecystitis/cholelithiasis (D98) |
| Ophthalmology |
| Retinopathy (F83) |
| Macular degeneration (F84) |
| Cataract (F92) |
| Glaucoma (F93) |
| Blindness (F94) |
| Otorhinolaryngology |
| Vertiginous syndrome (H82) |
| Presbyacusis (H84) |
| Deafness (H86) |
| Deafness (H86) |
| Hypertrophy tonsils/adenoids (R90) |
| Cardiology |
| Congenital anomaly cardiovascular (K73) |
| Ischemic heart disease with angina (K74) |
| Acute myocardial infarction (K75) |
| Ischemic heart disease without angina ((K76) |
| Heart failure (K77) |
| Atrial fibrillation/flutter (K78) |
| Paroxysmal tachycardia (K79) |
| Cardiac arrhythmia nos (K80) |
| Heart/arterial murmur nos (K81) |
| Pulmonary heart disease (K82) |
| Heart valve disease nos (K83) |
| Heart disease other (K84) |
| Hypertension uncomplicated (K86) |
| Hypertension complicated (K87) |
| Postural hypotension (K88) |
| Neurology |
| Transient cerebral ischemia (K89) |
| Stroke/cerebrovascular accident (K90) |
| Cerebrovascular disease (K91) |
| Neurological infection other (N73) |
| Malignant neoplasm nervous system (N74) |
| Benign neoplasm nervous system (N75) |
| Congenital anomaly neurological (N85) |
| Multiple sclerosis (N86) |
| Parkinsonism (N87) |
| Epilepsy (N88) |
| Migraine (N89) |
| Cluster headache (N90) |
| Facial paralysis/Bell’s palsy (N91) |
| Trigeminal neuralgia (N92) |
| Carpal tunnel syndrome (N93) |
| Peripheral neuritis/neuropathy (N94) |
| Angiology |
| Atherosclerosis/peripheral vascular disease (K92) |
| Pulmonary embolism (K93) |
| Phlebitis/thrombophlebitis (K94) |
| Varicose veins of leg (K95) |
| Rheumatology |
| Neck syndrome (L83) |
| Back syndrome without radiating pain (L84) |
| Acquired deformity of spine (L85) |
| Back syndrome with radiating pain (L86) |
| Rheumatoid/seropositive arthritis (L88) |
| Osteoarthrosis of hip (L89) |
| Osteoarthrosis of knee (L90) |
| Osteoarthrosis other (L91) |
| Shoulder syndrome (L92) |
| Tennis elbow (L93) |
| Osteoporosis (L95) |
| Psychiatry |
| Chronic alcohol abuse (P15) |
| Dementia (P70) |
| Organic psychosis other (P71) |
| Schizophrenia (P72) |
| Affective psychosis (P73) |
| Anxiety disorder/anxiety state (P74) |
| Somatization disorder (P75) |
| Depressive disorder (P76) |
| Neurasthenia/surmenage (P78) |
| Phobia/compulsive disorder (P79) |
| Personality disorder (P80) |
| Hyperkinetic disorder (P81) |
| Post-traumatic stress disorder (P82) |
| Mental retardation (P85) |
| Anorexia nervosa/bulimia (P86) |
| Psychosis nos/other (P98) |
| Pulmonology |
| Malignant neoplasm bronchus/lung (R84) |
| Malignant neoplasm respiratory other (R85) |
| Hypertrophy tonsils/adenoid (R90) |
| Chronic obstructive pulmonary disease (R95) |
| Asthma (R96) |
| Dermatology |
| Malignant neoplasm of skin (S77) |
| Dermatitis seborrhoeic (S86) |
| Dermatitis/atopic eczema (S87) |
| Psoriasis (S91) |
| Endocrinology |
| Malignant neoplasm thyroid (T71) |
| Goiter (T81) |
| Obesity (T82) |
| Overweight (T83) |
| Hyperthyroidism/thyrotoxicosis (T85) |
| Hypothyroidism/myxedema (T86) |
| Diabetes insulin dependent (T89) |
| Diabetes non-insulin dependent (T90) |
| Gout (T92) |
| Lipid disorder (T93) |
| Nephrology |
| Malignant neoplasm of kidney (U75) |
| Glomerulonephritis/nephrosis (U88) |
| Gynecology |
| Infertility/subfertility female (W15) |
| Pelvic inflammatory disease (X74) |
| Malignant neoplasm cervix (X75) |
| Malignant neoplasm breast female (X76) |
| Malignant neoplasm genital female other (X77) |
| Urology |
| Malignant neoplasm prostate (Y77) |
| Malignant neoplasm male genital other (Y78) |
| Benign prostatic hypertrophy (Y85) |
| Malignant neoplasm of kidney (U75) |
| Malignant neoplasm of bladder (U76) |
| Malignant neoplasm urinary other (U77) |
| nos=not otherwise specified.* Chronic health conditions were defined based on the ICPC-2 classification system using the concept of O’Halloran et al. [3].^†^A chronic health condition could be assigned to two specialties if commonly treated by both (Malignant neoplasm bronchus/lung treated by pulmonology and oncology). |
